# Castle on a cloud: endoscopic resection of a sessile serrated lesion overlying a colonic lipoma

**DOI:** 10.1093/jcag/gwae004

**Published:** 2024-02-07

**Authors:** Fiona Milne, Tao Wang, Robert Bechara

**Affiliations:** Division of Gastroenterology, Kingston Health Sciences Centre, Queen’s University, Kingston, Ontario K7L 2V7, Canada; Department of Pathology, Kingston Health Sciences Centre, Queen’s University, Kingston, Ontario K7L 2V7, Canada; Division of Gastroenterology, Kingston Health Sciences Centre, Queen’s University, Kingston, Ontario K7L 2V7, Canada

An asymptomatic 58-year-old male with coronary artery disease and atrial fibrillation was seen in endoscopy after positive faecal immunochemical test. Colonoscopy showed a 1.5 cm Paris Is + IIc polyp at the hepatic flexure with perceived high-risk features concerning for invasion. A therapeutic endoscopist performed a repeat exam for potential endoscopic resection. A magnifying exam revealed a JNET I lesion consistent with a sessile serrated lesion (SSL). Macroscopic exam was suspicious for a submucosal lesion beneath the SSL (Figure 1A–C). This was injected submucosally with saline and methylene blue, incised using the snare tip, with subsequent submucosal dissection again with the snare tip to ensure that the submucosal lesion was not arising from the muscularis propria. The submucosal lesion appeared to be a lipoma arising in the submucosa (Figure 1D). The lesion was removed enbloc (Figure 1E). Pathology was consistent with SSL overlying submucosal adipose tissue consistent with lipoma (Figure 1F).

**Figure 1.**
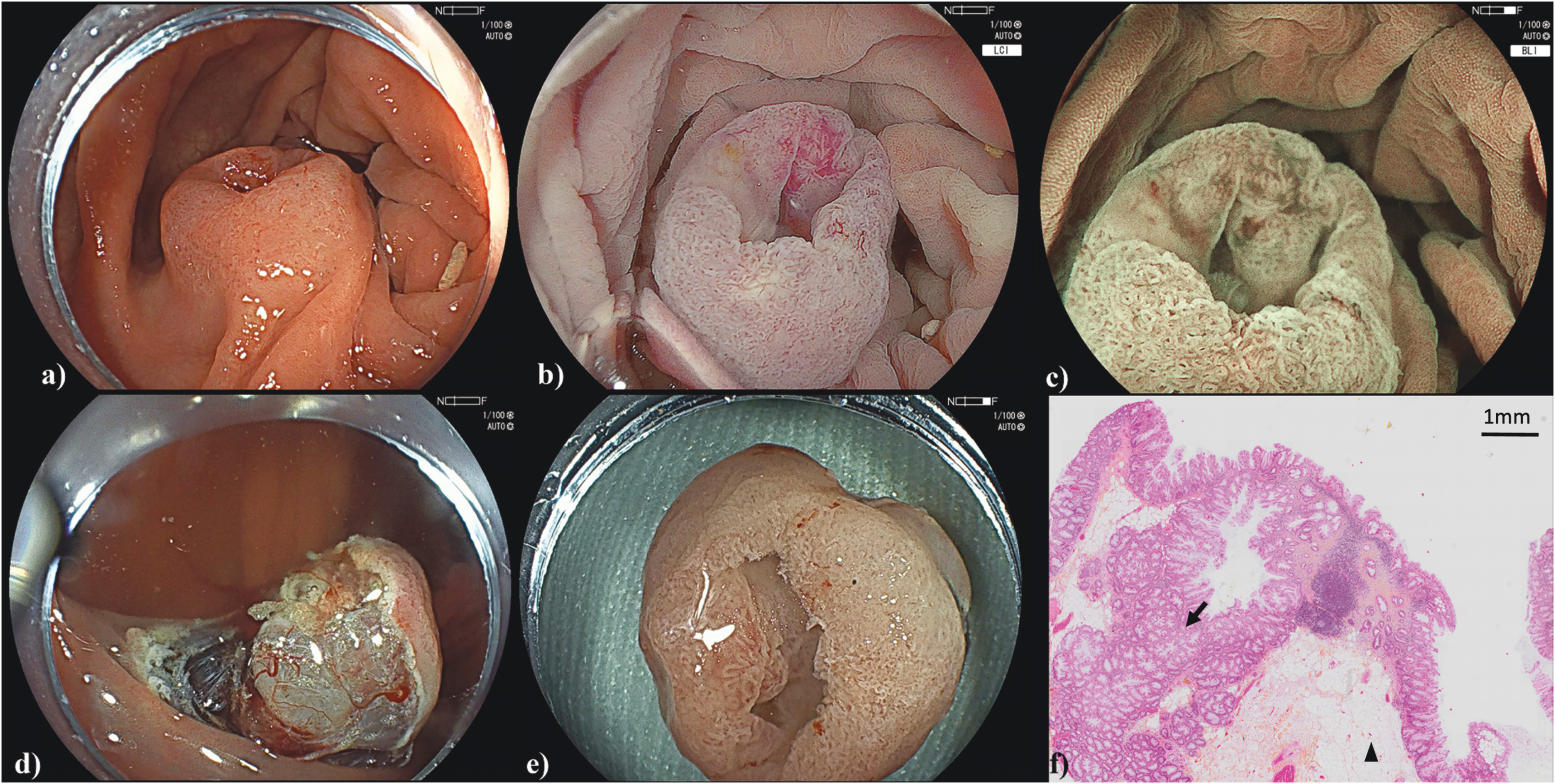
(A–C) Lesion under white light, linked colour imaging, and magnifying blue light imaging, respectively. (D) Partially dissected lesion with submucosal lipoma, (E) final specimen, and (F) lesion with haematoxylin–eosin–saffron stain under magnification (scale bar provided). Arrowhead denoting adipose tissue and arrow denoting SSL.

Lipomas are benign lesions that can be found throughout the gastrointestinal tract. They are most frequently found in the colon, particularly the right colon.^1^ They classically have a yellow ovoid appearance and are typically incidental findings during endoscopy. If they are large (greater than 4 cm), symptoms can develop including gastrointestinal bleeding, obstruction, and intussusception.^1^ It is rare to see associated mucosal abnormalities, but there are cases described of hyperplastic, sessile, and adenomatous polypoid tissue on top of lipomas.^2–4^ We describe a case of a serrated lesion overlying a right-sided colonic lipoma with an atypical appearance of a subepithelial lesion with central depression.

## Data Availability

There are no data associated with this manuscript.
